# Hybrid Perovskite Terahertz Photoconductive Antenna

**DOI:** 10.3390/nano11020313

**Published:** 2021-01-26

**Authors:** Petr A. Obraztsov, Vladislava V. Bulgakova, Pavel A. Chizhov, Alexander A. Ushakov, Dmitry S. Gets, Sergey V. Makarov, Vladimir V. Bukin

**Affiliations:** 1 Prokhorov General Physics Institute of the Russian Academy of Sciences, 119991 Moscow, Russia; vbulgakova573@gmail.com (V.V.B.); pvch@inbox.ru (P.A.C.); ushakov.aleksandr@physics.msu.ru (A.A.U.); vladimir.bukin@gmail.com (V.V.B.); 2 Department of Physics and Engineering, ITMO University, 197101 St. Petersburg, Russia; dmitry.gets@metalab.ifmo.ru (D.S.G.); s.makarov@metalab.ifmo.ru (S.V.M.)

**Keywords:** perovskite, hybrid organic–inorganic perovskite, terahertz, terahertz emission, photoconductive antenna, pump–probe spectroscopy, LAPCA, MAPbI_3_, MAPbBr_3_

## Abstract

Hybrid organic–inorganic perovskites, while well examined for photovoltaic applications, remain almost completely unexplored in the terahertz (THz) range. These low-cost hybrid materials are extremely attractive for THz applications because their optoelectronic properties can be chemically engineered with relative ease. Here, we experimentally demonstrate the first attempt to apply solution-processed polycrystalline films of hybrid perovskites for the development of photoconductive terahertz emitters. By using the widely studied methylammonium-based perovskites MAPbI_3_ and MAPbBr_3_, we fabricate and characterize large-aperture photoconductive antennas. The work presented here examines polycrystalline perovskite films excited both above and below the bandgap, as well as the scaling of THz emission with the applied bias field and the optical excitation fluence. The combination of ultrafast time-resolved spectroscopy and terahertz emission experiments allows us to determine the still-debated room temperature carrier lifetime and mobility of charge carriers in halide perovskites using an alternative noninvasive method. Our results demonstrate the applicability of hybrid perovskites for the development of scalable THz photoconductive devices, making these materials competitive with conventional semiconductors for THz emission.

## 1. Introduction

Hybrid organic–inorganic perovskites are a novel type of semiconductors demonstrating great potential for low-cost and high-performance solar cell application. The energy conversion efficiency of perovskite solar cells has skyrocketed from 3.8% to >25% [1,2,3,4] in less than 10 years and now rivals the level of heavily developed conventional CIGS-, CdTe-, and Si-based solar cells. Both experimental measurements and theoretical calculations show that MAPbI_3_ and related materials, such as MAPbI_3_−xBrx (MAPbIBr_3_), have bandgaps in the range of 1.1–2.1 eV, corresponding to the visible light region [5]. Their optical absorption strength is comparable to other classic semiconductors, such as GaAs, InP, and CdTe. These hybrid materials also show relatively high and balanced electron and hole mobility and very fast electron–hole pair generation. In addition, the carrier diffusion length is more than 1 mm, implying a low concentration of deep defects [6].

The basic properties of perovskites that make them attractive for solar cells also make them appealing for terahertz (THz) range applications [7,8]. Recent experiments on the generation of ultrafast photocurrents in halide perovskites excited with a femtosecond laser leading to emission of THz pulses have demonstrated the potential of these hybrid materials for THz optoelectronics [9,10,11,12,13,14,15]. However, while well examined in the optical range [16], hybrid perovskites remain almost completely unexplored for THz applications. Meanwhile, improvement of the efficiency of THz pulse sources and other THz optoelectronic devices with better designs or novel materials is one of the major goals of ongoing research in this field [17,18].

Here, we experimentally demonstrate the applicability of solution-processed polycrystalline films of the hybrid perovskites MAPbI_3_ and MAPbBr_3_ for the development of large-aperture photoconductive antennas (HP-LAPCAs).

Photoconductive antennas are promising sources of THz radiation due to their compactness, relatively high power, and narrow directivity of the output radiation [19]. Moreover, LAPCAs are unique sources of quasi half-cycle (unipolar) THz pulses covering the lower part of the THz spectrum ranging from 0.1 to 1 THz suitable for experiments on the acceleration of charged particles and electrons [20,21].

The mechanism of THz generation from a biased photoconductive antenna can be understood as follows: a femtosecond pulsed laser illuminates the gap of a semiconductor material and creates excited carriers in the conduction band. An external bias field accelerates the carriers up to the saturation velocity, leading to a transient photocurrent. This transient current in picosecond time domain is responsible for the THz pulse emission. In the far field, the THz electric field is proportional to the time derivative of the transient current, which can be expressed as E(*t*)~d*J*/d*t*. Current density can be written as J(*t*) = N(*t*)μEb, where N(*t*) is the number of charge carriers, μ is the mobility of the charge carrier, and Eb is the applied electric field. Therefore, the efficiency and spectral characteristics of the THz source crucially depend on the lifetime and the achievable concentration of the excited carriers in the material.

In this report, we perform systematic investigations of ultrafast carrier dynamics in organic–inorganic halide perovskites and study the properties of THz radiation emitted from photoconductive antennas on their base. The work presented here examines polycrystalline perovskite films excited both above and below the bandgap, as well as the scaling of the THz emission with the applied bias field and the optical excitation fluence. The motivation for this work is to determine the suitability of hybrid perovskite LAPCAs for the efficient generation of THz pulses. We also compare the THz radiation from polycrystalline MAPbI_3_ and MAPbBr_3_ antennas to explicitly demonstrate that the variety of solution-processed hybrid perovskites holds a strong potential as easily scalable and chemically tunable materials for THz emitters. Moreover, the conducted studies allowed us to reveal the still-debated carrier lifetime and mobility of charge carriers in widely studied perovskites using an alternative noninvasive method. The developed method can be further applied to study the excited carrier transport in the variety of 3D and 2D materials having a perovskite structure.

## 2. Materials and Methods

### 2.1. Perovskite Synthesis

The synthesis from solutions containing perovskite precursors was performed using well-developed procedures [22]. Perovskite precursors and a dimethyl formamide (DMF) solvent were purchased from suppliers and used as received. The samples studied in the current work were synthesized using the following reagents: lead(II) iodide (99.99%, trace metals basis), TCI; methylammonium iodide (99.8%), Dyesol; lead(II) bromide (99.99%, trace metals basis), TCI; methylammonium bromide (99.8%), Dyesol. Glass substrates of 1.5 × 1.5 cm^2^ size were subsequently ultrasonicated in acetone and 2-propanol for 5 min, respectively, then washed with deionized water, dried with dry air, and finally cleaned with O_3_ for 10 min.

Preparation of MAPbBr_3_ solution: PbBr_2_ (293.6 mg) and MABr (89.57 mg) were subsequently dissolved in 1 mL of mixture of anhydrous DMF and DMSO (7:3 ratio by volume). Preparation of MAPbI_3_ solution: PbI_2_ (461.01 mg) and MAI (158.97 mg) were subsequently dissolved in 1 mL of mixture of anhydrous DMF and DMSO (9:1 ratio by volume). All the solutions were prepared in an N_2_-filled glove box operating at 0.1 ppm of both O_2_ and H_2_O.

### 2.2. Thin Film Deposition

The spin-coating cycle had the following steps: At the first step, the spin coater reached a rotational speed of 500 rpm for 5 s and kept that speed for 20 s; then it accelerated up to 3000 rpm for 3 s and kept this speed for 17 s. At the third second of the second cycle (sixth second for MAPbBr_3_), 0.5 mL of anhydrous diethyl ether was dripped onto the substrate. The films were annealed on the hot plate at 50 °C for 1 min and then at 100 °C for 10 min.

**Figure 1:**
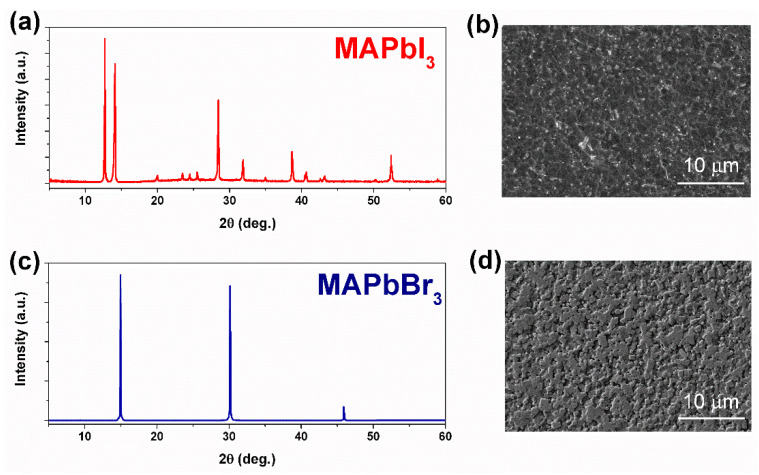
Typical XRD spectra and SEM images of fabricated polycrystalline MAPbI_3_ (**a**,**b**) and MAPbBr_3_ (**c**,**d**) films.

The morphology of the prepared polycrystalline perovskite films was characterized using X-ray diffraction (XRD) and scanning electron microscopy (SEM) methods. Figure 1 shows the typical XRD spectra and SEM images of synthesized MAPbI_3_ and MAPbBr_3_ films.

### 2.3. Materials Characterization

#### 2.3.1. Steady-State Properties

The linear optical absorption measurements were done using a two-channel PerkinElmer Lambda 950 spectrophotometer. To study the photoluminescent (PL) properties, we used an integrating sphere with a sample placed in the sphere center at 45 degrees relative to the incident laser beam. As a source, we used a semiconductor laser diode with a wavelength of 405 nm and a spot size of approximately 0.2 cm at a level of 1/e.

#### 2.3.2. Optical Pump/THz Probe

In optical pump/THz probe experiments, the output beam of a Ti:sapphire regenerative amplifier (Spectra-Physics Tsunami+, USA, Spitfire Pro 35—XP, 800 nm fundamental wavelength, 2.9 mJ pulse energy, 40 fs pulse duration, 1 kHz repetition rate, ) was split into two arms with a 70/30 beam splitter. A major portion of the laser beam was used to pump the LiNbO_3_ THz source. Here, we employed a well-known scheme for the generation of high-power coherent terahertz radiation by optical rectification of tilted-intensity-front femtosecond pulses in LiNbO_3_ nonlinear crystal [23]. The output laser-driven THz beam (used as a probe beam) was collimated and focused with polytetrafluoroethylene (PTFE) lens on the central part of the perovskite film with an intensity spot size of ~1 mm. A minor portion of the laser beam was directed to the sample surface without focusing to excite the perovskite film optically. The laser and THz beams were overlapping on the sample. The time delay between the optical and THz pulses at the sample position was controlled with a motorized delay line introduced in the pump optical arm. An average power of THz pulses transmitted through the optically excited perovskite sample was measured with a Golay cell detector (Tydex GC-1P, St.Petersburg, Russia). Therefore, monitoring the Golay cell output as a function of delay time between pump and probe pulses provided tracking of the temporal dynamics of pump-induced changes in the studied samples.

#### 2.3.3. Optical Pump/Optical Probe

To perform the time-resolved measurements in the optical range and to reveal the relaxation rates of photoexcited carriers in perovskite MAPbI_3_ and MAPbB_r3_ films, we employed a multicolor transient absorption pump–probe setup. A detailed description of the method and setup we reported previously can be found elsewhere [24,25]. The femtosecond pulses with central wavelengths of 800 and 400 nm (SHG) and a femtosecond continuum were employed as pump and probe, respectively. The pump pulses were delivered by a Ti:sapphire regenerative femtosecond amplifier (800 nm wavelength, 40 fs pulse duration, 1 kHz repetition rate). The probe continuum pulses were generated by focusing the beam (at a wavelength of 800 nm and with an average power of 100 mW) on a sapphire crystal. The diameter of the pump beam at the sample surface was about 500 μm. The femtosecond continuum was used to probe the differential transmission (ΔT/T) in a wide spectral range spanning from 380  to 900 nm. The pump and probe beams were polarized collinearly. A rapid motorized delay line controlled the time delay between the pump and the probe pulses. The pump-induced change of the probe transmission was detected with a spectrometer equipped with a Si CCD array (CDP ExciPro 2012). All measurements were performed at room temperature in ambient conditions.

### 2.4. HP-LAPCA Fabrication

To evaluate the performance of synthesized polycrystalline hybrid perovskites as terahertz emitters, we deposited parallel aluminum stripe-line electrodes on top of synthesized films. The gap between the electrodes for different devices varied from 2 to 4 mm, while the width of the electrodes was 12 mm. The perovskite thin film with conductive electrodes forms the simple-geometry large-aperture photoconductive antenna, where the perovskite material serves as a light-absorbing photoconductive substrate, while the electrodes are used to apply bias high voltage accelerating the generated photocarriers.

### 2.5. THz Emission Experiments

**Figure 2:**
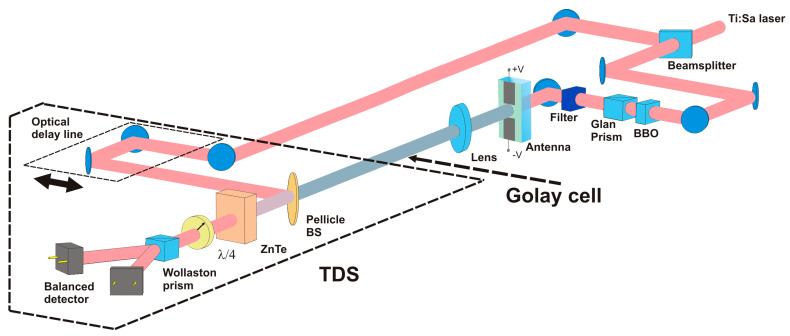
Experimental setup for the generation and detection of terahertz (THz) pulses from a large-aperture photoconductive antenna.

In THz emission experiments, the photoconductive antenna was excited by a femtosecond Ti:sapphire laser (Coherent Legend Elite, Santa Clara, CA, USA, 1 kHz repetition rate, 130 fs duration, 800 nm center wavelength, beam diameter of 10 mm at 1/e^2^). The antenna was excited at the fundamental (800 nm) and second harmonics (400 nm) corresponding to pump photon energies of 1.5 and 3 eV correspondingly. To generate the second harmonic, a BBO crystal (I-type, thickness of 200 μm) was used (the energy of the second harmonic pulses was 0.3 mJ). A Glan prism polarizer was used to change the energy of an optical pulse, and an optical filter was used to absorb radiation at the fundamental harmonic. Optical pumping of the perovskite part of the antenna was performed in transmission geometry from the side of the glass substrate (as shown in Figure 2).

Bias high-voltage pulses with amplitudes of up to Ub = 3 kV, a duration of ~10 ns, and a repetition rate of 500 Hz were applied to the electrodes. The repetition rate of the high-voltage pulses was 500 Hz to increase signal to noise ratio SNR at a frequency of 1 kHz [26].

The terahertz waveforms were obtained using a typical time-domain spectroscopy (TDS) technique. In these experiments, the optical beam was split into two parts with a beam splitter (BS). The main part of optical radiation passes through the BS and is used to generate THz radiation in the antenna. Part of the radiation reflected from the BS entered the delay line for detecting the THz waveform. The THz radiation beam was focused by a PTFE lens (f = 100 mm) on an electro-optical ZnTe crystal (0.5 mm thick, [110]—cut). The temporal dependence of the electric field strength of the terahertz pulse was recorded by a balanced detector with a 500 Hz triggered lock-in amplifier. To analyze the energy of the generated THz pulses, the Golay cell was used as a detector. In these experiments, laser radiation was gated at a frequency of 10 Hz. All measurements were performed at room temperature in ambient conditions. The experimental setup is schematically shown in Figure 2.

## 3. Experimental Results and Discussion

### 3.1. Steady-State Properties

**Figure 3:**
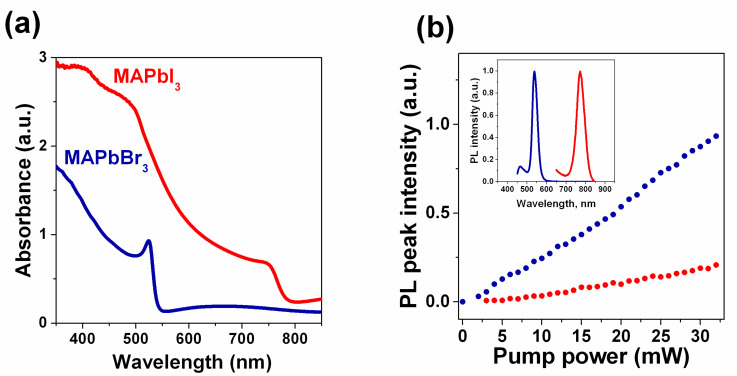
Steady-state optical properties of synthesized polycrystalline MAPbI_3_ and MAPbBr_3_ films placed on glass substrates. (**a**) Optical transmission spectra; inset shows the typical SEM image of an MAPbI_3_ film; (**b**) photoluminescence spectra obtained under excitation with a 400 nm laser diode; (**b**) the dependence photoluminescence (PL) peak intensity on pump power. Blue and red colors correspond to MAPbBr_3_ and MAPbI_3_ samples, respectively. Inset demonstrates the corresponding normalized PL spectra.

The steady-state optical properties of the fabricated polycrystalline thin films with a thickness of ~600 nm were characterized by optical absorption and photoluminescence spectroscopies. Figure 3a shows the visible-range absorption spectra of the fabricated MAPbI_3_ and MAPbBr_3_ films. The photoluminescence spectra obtained from MAPbI_3_ and MAPbBr_3_ excited with a 400 nm CW diode laser and the dependences of the PL peak intensity on pump fluence are shown in Figure 3b.

### 3.2. Ultrafast Dynamics

In order to study the dynamic response of synthesized perovskite materials and to reveal the characteristic relaxation dynamics/carrier lifetime, we performed femtosecond laser-driven optical pump/THz probe and optical pump/optical probe measurements.

**Figure 4:**
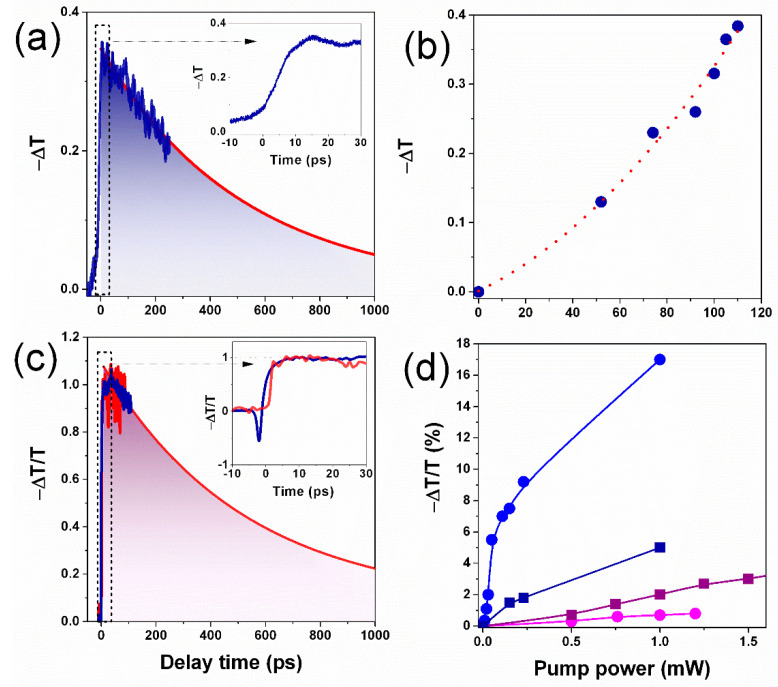
Temporal dynamics of the pump-induced THz/visible range transmission change in MAPbI_3_ (blue) and MAPbBr_3_ (red) films. (**a**) THz transmission change (ΔT) as a function of delay time between 800 nm pump and THz probe pulses. (**c**) Optical transmission change (ΔT/T) as a function of delay time between 400 nm pump and 500 nm probe 40 fs pulses. Exponential fitting with a decay constant τ = 360 ps is shown with solid red lines. (**b**,**d**) The transmission change amplitude as a function of the pump–pulse power. The data obtained under 400 and 800 nm excitations are shown with blue and magenta colors correspondingly.

The typical dynamics of an 800 nm pump-induced THz transmission change (ΔT) in 600 nm thick polycrystalline MAPbI3 is shown in Figure 4a. In Figure 4a, one can clearly see that at zero delay, the optical pumping of the sample leads to instantaneous decrease of the sample THz transmission related to fast photoexcited carrier generation. The THz absorption for a thin film is typically proportional to the photoconductivity (Δσ), which in turn is proportional to the photogenerated carrier density (the number of excited carriers) (N) and mobility (μ) following E~Eσ~μeN [27].

The relaxation dynamics of the transmission change following the photoexcitation provides an estimation of the carrier lifetime in the studied material. The decay constant τ = 360 ps derived from the exponential fitting of the experimental data is in agreement with the previously reported data on the carrier lifetime in halide perovskites [28]. Figure 4b shows the pump-induced transmission change (ΔT) as a function of pump fluence.

To reveal the relaxation rates of photoexcited carriers in perovskite films, we employed a multicolor transient absorption pump–probe setup based on the same Ti:sapphire femtosecond laser as used in optical pump/THz probe experiments. A detailed description of the method and setup can be found in the methods section. The normalized differential transmission of a 500 nm probe as a function of delay time between pump and probe pulses measured for both samples is presented in Figure 4b. Under both above and below the bandgap, optical pumping of the decreased transmission (pump-induced absorption) of the probe pulse at a wavelength of 500 nm can be observed. The observed transmission change is associated with the reabsorption of pump photons by the pump-induced excited carriers [29,30]. Therefore, the temporal dynamics and pump fluence dependence of the induced absorption provide direct information on the lifetime and created a concentration of excited carriers. Figure 4b shows the pump fluence dependence of ΔT measured at zero delay time for above and below the bandgap excitation of MAPbI_3_ and MAPbBr_3_ samples. Reasonably at lower pump fluences, corresponding to lower carrier concentrations, ΔT linearly depends on pump fluence, which is in accordance with the optical pump/THz probe data (see Figure 4a). However, at a higher pump fluence, ΔT clearly demonstrates the saturation behavior. Moreover, for both samples, absorption of pump photons having energy exceeding the bandgap of the material leads to the creation of a larger number of carriers comparing with below the bandgap excitation. Note that under 800 nm excitation, we do not observe a significant contribution of two-photon processes.

### 3.3. THz Emission from HP-LAPCA

**Figure 5:**
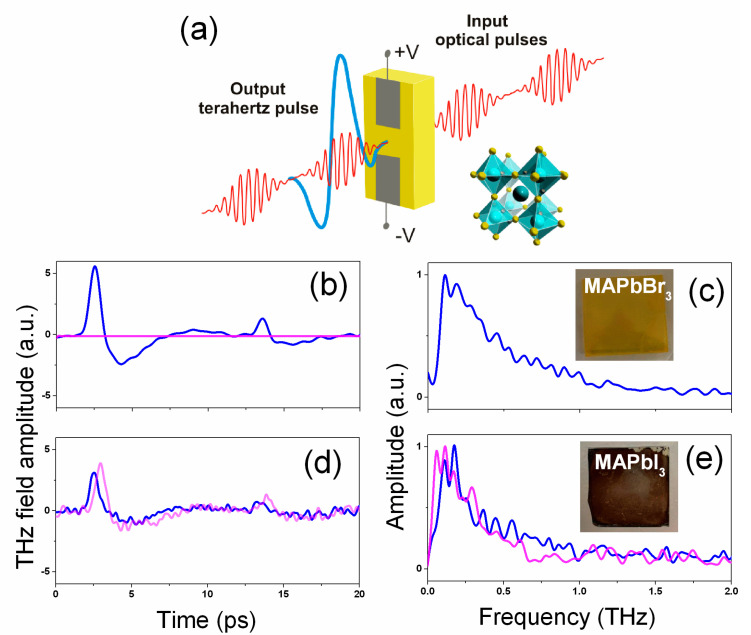
(**a**) Scheme of the THz emission from an HP-LAPCA (large-aperture photoconductive antenna). Time domain waveforms of the THz emission from MAPbI_3_ (**b**) and MAPbBr_3_ (**d**) and corresponding frequency domain spectra (**c**,**e**) obtained under above and below the bandgap excitation. The data obtained under 400 and 800 nm excitations are shown in blue and magenta colors.

In the THz emission experiments, optical pumping of the perovskite part of the antenna was performed in transmission geometry from the side of the glass substrate in a way schematically shown in Figure 5a. Figure 5b–e shows the typical waveforms and corresponding frequency (FFT) spectra obtained from the fabricated LAPCAs based on MAPbI_3_ and MAPbBr_3_ perovskites under 400 and 800 nm optical excitations.

Under 400 nm optical excitation from both materials, quasi-unipolar THz pulses with an full width at half maximum (FWHM) duration of ~1 ps were obtained. Under 800 nm excitation, THz pulses with similar waveforms and spectra were also obtained from an MAPbI_3_-based LAPCA. The obtained waveforms are typical for photoconductive antennas with a large interelectrode distance [19]. It is important to note that in such geometry of emitter, the temporal profile of the THz pulse is determined by the interelectrode distance rather than by the lifetime of the carriers in the photoconductive substrate.

As described in detail in the methods section, the energy of the generated THz pulses was measured with a Golay cell detector. The assumption of non-uniform illumination with a Gaussian intensity profile optical beam was used to analyze the data. The THz pulse energy was calculated using the following equation:

(1) $$W_{\mathrm{THz}}=\beta \underset{S}{\int }F_{\mathrm{THz}}\left(r\right)\mathrm{dS}$$

where F_THz_ is terahertz fluence: F_THz_(*r*) = F(r)·η(F(r)), and F(r) = (2W_opt_/πr_0_^2^)·exp(−2r^2^/r_0_^2^) is optical fluence [31]. The optical-to-THz conversion efficiency is η = (τE_b_^2^/2F(r)Z_0_)·(F(r)/F(r) + F_sat_)^2^, where τ is the THz pulse duration, E_b_ is the electric bias field, Z_0_ is the free space impedance, F_sat_ = h*ν*·(1 + n)/eµZ_0_(1 − R) is the necessary fluence for extracting half of the maximum radiated field (saturation fluence), h*ν* is the photon energy of the excitation wavelength, e is the electron charge, n is the THz refractive index, µ is the carrier mobility, and R is the optical reflection of the semiconductor substrate. The THz pulse duration in calculations is τ = 1 ps, and the size of the 10 mm optical beam diameter at a 1/e^2^ level. Integration is carried out over the area between the electrodes. The dimensionless factor *β* is included in Equation (1) to take into account the attenuation of the THz pulse energy due to absorption in the antenna substrate, reflections, and so forth. We assume that the propagation medium of the THz-generated pulse is air due to the fact that the perovskite layer (600 nm) is small on the scale of the terahertz wavelength.

**Figure 6:**
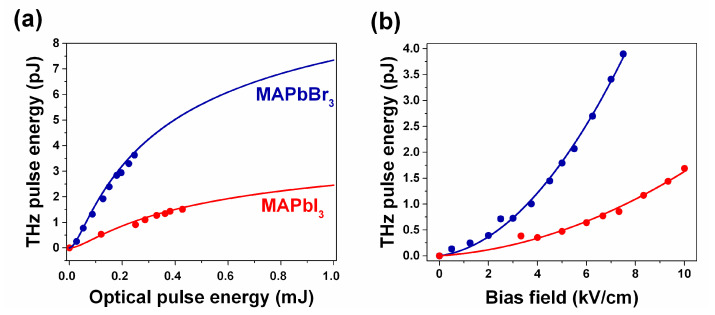
The terahertz pulse energy as a function of the optical excitation energy (**a**) and applied bias voltage (**b**) obtained from the HP-LAPCA optically excited above the bandgap (400 nm). The data obtained for HP-LAPCAs based on MAPbI_3_ and MAPbBr_3_ are shown with red and blue colors correspondingly.

Figure 6 shows a comparison of the data obtained for MAPbI_3_ and MAPbBr_3_ under 400 nm excitation. We found that in both cases, the LAPCA’s emitted terahertz energy is quadratically dependent on the applied bias field (Eb). Lower relative permittivity in combination with higher exciton energy in MAPbBr_3_ in comparison with MAPbI_3_ in turn may lead to higher efficiency of the optical-to-THz conversion efficiency.

The experimental data are in good agreement with the theory [32]. Using the above data and Equation (1), we estimate the saturation fluence for both perovskite materials: F_sat_ = 0.194 mJ/cm^2^ and F_sat_ = 0.293 mJ/cm^2^ for MAPbBr_3_ and MAPbI_3_ correspondingly. Therefore, the carrier mobilities derived from the THz emission experiments are µ = 58 cm^2^/V·s and µ = 88 cm^2^/V·s for MAPbI_3_ and MAPbBr_3_. The obtained charge carrier mobilities are in agreement with the data obtained for the studied perovskite materials using conventional methods [28].

**Figure 7:**
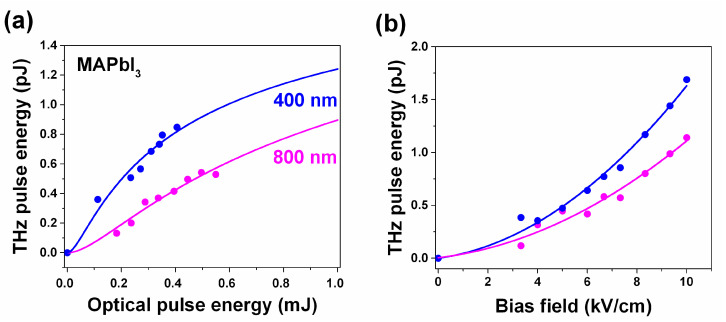
The MAPbI_3_-based HP-LAPCA terahertz pulse energy as a function of the optical excitation energy (**a**) and applied bias voltage (**b**) for pumping above (400 nm, blue) and below (800 nm, magenta) the bandgap.

We assume that the absorption coefficients for MAPbI_3_ are 0.94 at 400 nm and 0.22 at 800 nm (Figure 3a). Figure 7 shows a comparison of the THz pulse energy and the optical energy and applied bias voltage obtained from the MAPbI_3_-based LAPCA under above and below the bandgap excitation. In accord with our optical pump/optical probe data in the case of 800 nm, there is a considerably lower carrier generation since the excitation energy is well below the bandgap of both materials. Moreover, under 800 nm excitation of the MAPbBr_3_ LAPCA, we were not able to generate any detectable THz signals.

## 4. Conclusions

To summarize, the applicability of solution-processed polycrystalline films of hybrid organic–inorganic perovskites for the development of terahertz radiation emitters is demonstrated. The large-aperture photoconductive antennas fabricated using methylammonium-based perovskites demonstrate a generation of several picojoule THz pulses under above and below the bandgap optical excitation. These results make hybrid perovskite materials competitive with conventional semiconductors for THz emission. Moreover, by applying the combination of time-resolved pump–probe spectroscopy and THz emission experiments, we determine a ~360 ps carrier lifetime and mobilities of µ = 58 cm^2^/V·s and µ = 88 cm^2^/V·s in MAPbI3 and MAPbBr3 polycrystalline films, correspondingly. The developed method can be further applied to study the variety of 3D and 2D materials having a perovskite structure.
